# Morphological and Transcriptomic Analyses Reveal the Involvement of Key Metabolic Pathways in Male Sterility in *Chimonanthus praecox* (L.) Genotypes

**DOI:** 10.3390/plants13182571

**Published:** 2024-09-13

**Authors:** Bin Liu, Huafeng Wu, Yinzhu Cao, Xiaowen Zheng, Haoxiang Zhu, Shunzhao Sui

**Affiliations:** 1 Chongqing Engineering Research Center for Floriculture, Key Laboratory of Agricultural Biosafety and Green Production of Upper Yangtze River (Ministry of Education), College of Horticulture and Landscape Architecture, Southwest University, Chongqing 400715, China; 15705983137@163.com (B.L.); swuwhf@126.com (H.W.); yinzhu202108@163.com (Y.C.); wennnn_n@163.com (X.Z.); 2 College of Horticulture and Landscape Architecture, Southwest University, Chongqing 400715, China; zhuhx8910@swu.edu.cn

**Keywords:** *Chimonanthus praecox*, male sterility, transcriptome, metabolic pathways

## Abstract

*Chimonanthus praecox* (Calycanthaceae family) is a unique ornamental and economic flowering tree in China, and after thousands of years of cultivation, it has produced several varieties and varietal types. Notably, male sterility is common in flowering plants and is an important tool for the genetic improvement in plants and optimization using hybrid plant technology; however, there have been no reports on male-sterile material or related studies on *C. praecox*. To our knowledge, this is the first time that *C. praecox* male sterility is dissected unveiling the involvement of key metabolic pathways. Notably, male sterility in *C. praecox* was observed during the budding period and likely occurred during the premature stage of pollen cell maturation. Additionally, differentially expressed genes in the starch and sucrose metabolism pathway and the plant hormone signal transduction pathway showed regular expression trends. This study reports on significant genetic differences that contribute to male sterility in *C. praecox* and provides a basis for further research and breeding strategies.

## 1. Introduction

*Chimonanthus praecox* (Calycanthaceae family) is a traditional, valuable flowering tree native to China that blooms in winter and has a pleasant fragrance; it is popular and has been extensively introduced and cultivated globally [1,2]. The flowering period of *C. praecox* can be up to 5 months, from late November to March. It can be used as cut flower material in garden ornamentation, and it exhibits an extended vase life of over 3 weeks [3,4]. Additionally, in Chinese traditional medicine, *C. praecox* flowers are often used to treat cough, fever, rheumatoid arthritis, etc. Moreover, various bioactive substances, such as essential oils and alkaloids, have been extracted from different organs and tissues of *C. praecox* [5,6]. *C. praecox* has been cultivated for thousands of years, and several varieties and varietal types have been produced through an extensive cultivation process [7]. The level of diversity within a species determines its ability to adapt to environmental changes and its evolutionary potential [8].

Notably, the higher the genetic diversity, the greater the ability to withstand adverse environments; conversely, a lower genetic diversity is associated with the loss of species genes and the extinction of the species. The mating system, which refers to the proportion of selfing and heterosis, is an important factor affecting the genetic diversity and distribution patterns of plant populations [9]. Typically, seeds produced from heterosis often exhibit heterosis advantage due to pollen sources from different plants [10], primarily contributing to the higher genetic diversity of the heterozygous progeny [11]. For plants, the mating system involves how they produce their offspring and is crucial to their adaptation to the environment and species diversity. In a heterotic mating system, the reception of pollen from other plants via pollinators can increase genetic diversity, thereby enabling the conservation of the inheritance of genes, promoting the improvement in plant varieties, and enhancing the environmental adaptability of plants [12]. Approximately 75% of land plant species are hermaphroditic. Notably, intrafloral dichogamy at the single-flower level is extensive. Such phenomena have been found in plants of several families and genera, such as Magnoliaceae, Juglandaceae, Ranunculaceae, and Lauraceae [13,14,15,16]. Herkogamy in hermaphroditic flowers refers to the spatial separation of the male and female stamens, which can be categorized in various ways. Regardless of the herkogamy type, its primary function is to prevent self-fertilization and the functional interference of the reproductive organs of both sexes [17].

*C. praecox* varieties are primarily heterogamous, dichogamous, and herkogamous hermaphrodite flowers. Dichogamy is a floral mechanism in which the development of the pistil and stamen is not synchronized within the flower. In this phenomenon of *C. praecox*, the pistil matures first, with the stigma becoming receptive before the flower fully opens. As the perianth unfolds, the receptivity of the stigma gradually reduces. Typically, the anthers in the flower undergo dehiscence between the third and fifth day after the flower opens. Subsequently, the receptivity of the stigma is significantly reduced, affecting vitality. The herkogamy of *C. praecox* is primarily due to the growth of anthers dorsal to the stigma, which prevents the pollen from directly falling onto the stigma. Furthermore, during flower opening, stamens of *C. praecox* exhibit centrifugal outward spreading and centripetal aggregation movement, that is, anther spreading and anther erection surrounding the stigma in two phases, of which stamens with non-dehiscence anthers expose the stigma to promote heterogamous pollination, and anthers that undergo dehiscence before stamen erection facilitate pollination by insects, consequently protecting the stigma [18,19]. Notably, while heterosis pollination might be promoted through dichogamy or herkogamy, neither mechanism is as effective as limiting self-pollination through self-incompatibility or dioecism. Plant male sterility refers to the abnormal development of plant male organs, and male sterility allows breeders to eliminate the laborious process of self-mating and effectively utilize the advantages of hybridization, which greatly improves the efficiency of breeding [20]; however, there have been no reports on male sterility in *C. praecox* to date.

In this study, we used transcriptome sequencing technology to analyze the transcriptomes of abortive and normally developing anthers of *C. praecox* to investigate the molecular mechanism of anther abortion in *C. praecox*. We report for the first time the male sterility trait in *C. praecox* and identify the association of some differentially expressed genes involved in the starch and sucrose metabolism pathways and the plant hormone signal transduction pathways in different plant materials during the pollination process; this represents the basis for the study of the regulatory mechanism of male sterility in *C. praecox* and provides further reference into its mechanism. 

## 2. Results

### 2.1. Morphological Characterization and Paraffin Section Analysis

**Figure 1:**
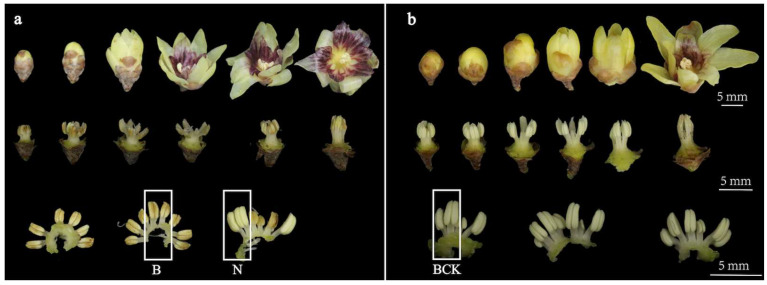
Morphological characteristics of different flowering stages of *C. praecox*: (**a**) morphology and androgynous characteristics of CP-MS at different flowering stages, where B is part of abortive stamens collected, and N represents normally developing stamens collected; (**b**) morphology and androgynous characteristics of CP-N at different flowering stages; BCK denotes a collection of normally developing stamens.

Phenotypic observations were made using CP-MS, a *C. praecox* anther-aborting variety, and CP-N, a *C. praecox* normal anther-developing variety. In the CP-MS, the vast majority of anthers aborted at the bud stage, and other tissues grew and developed normally (Figure 1). During pollination, the stamens of CP-MS exhibited lying and erect movements, and the perianth segments rolled back at full bloom, completely exposing the androecium. In CP-N, each floral organ grew and developed normally, with the anthers gradually undergoing abortion in the late bloom stage.

**Figure 2:**
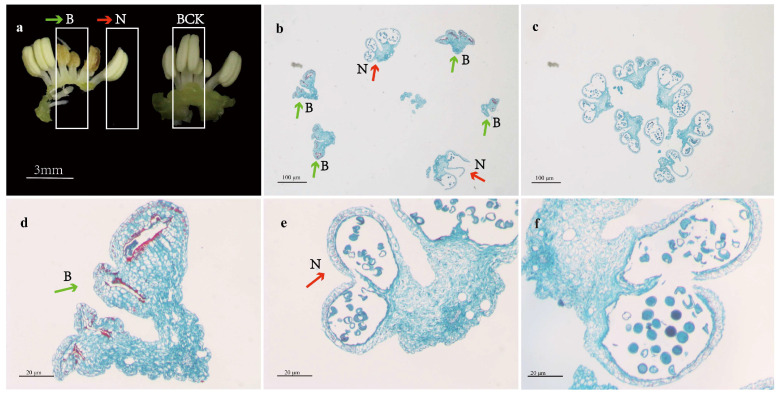
Paraffin sections of CP-MS and CP-N varieties: (**a**) morphological characteristics of CP-MS and CP-N varieties; (**b**) paraffin section of entire anther from CP-MS variety; (**c**) paraffin section of entire anther from CP-N variety; (**d**) paraffin section of individual sterile anther from CP-MS variety; (**e**) paraffin section of individual normal anther from CP-MS variety; (**f**) paraffin section of individual anther from CP-N variety. Note: B denotes sections from abortive stamens collected from CP-MS, N denotes sections from normal developing stamens collected from CP-MS, and BCK indicates sections from normal developing stamens collected from CP-N.

Through the analysis of paraffin sections, it was observed that CP-MS varieties exhibit two conditions of anther development, namely sterility and normal development, with a higher incidence of sterility than normal development. In sterile anthers, not only do the locules noticeably shrink inwardly, shrinking noticeably with pronounced lignification along the inner edges, but they also demonstrate a significant reduction in or absence of pollen grains. In contrast, anther and pollen grain development in CP-N varieties is generally normal (Figure 2).

### 2.2. Overview of Transcriptome Data and Functional Annotation Information

**Figure 3:**
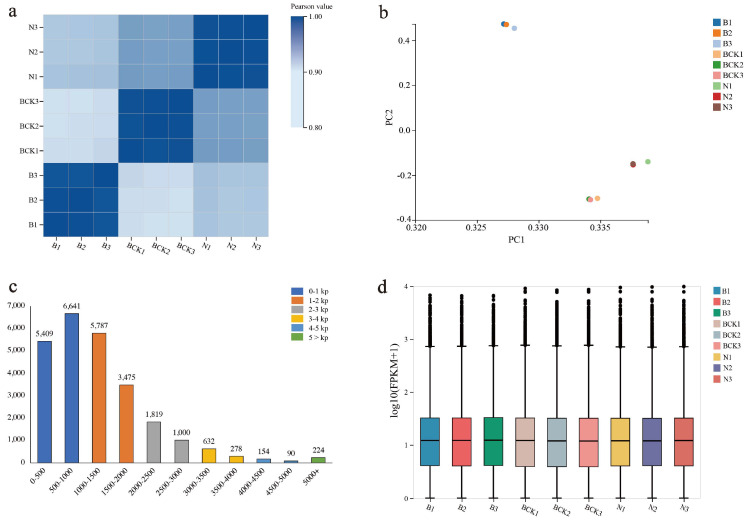
Overview of transcriptome data: (**a**) sample correlation heatmap; (**b**) principal component analysis; (**c**) transcript length distribution; (**d**) box–line plot of gene expression level distribution.

Nine samples were measured in this study (B, N, and BCK at the bud stage (Section 2.1), with three biological replicates each), and each sample produced an average of 6.62 Gb of data (Table S1), with a positive correlation between the samples (Figure 3a). Additionally, principal component analysis showed that several samples in the same group exhibited close relationships (Figure 3b). The average comparison rate of genome was 97.88% (Table S2). The average comparison rate for gene sets was 62.25% (Table S3). A total of 30,209 expressed genes, including 22,112 known and 8097 newly predicted genes corresponding to 15,736 new transcripts, were detected. The length of known gene transcripts was the highest in the range of 500–1000, with 6641 transcripts, followed by distributions in the range of 1000–1500 and 0–500, with 5787 and 5409 transcripts, respectively (Figure 3c and Table S4). The number of genes with expression levels ≥10 in different samples accounted for approximately 50% of the total (Figure 3d).

**Figure 4:**
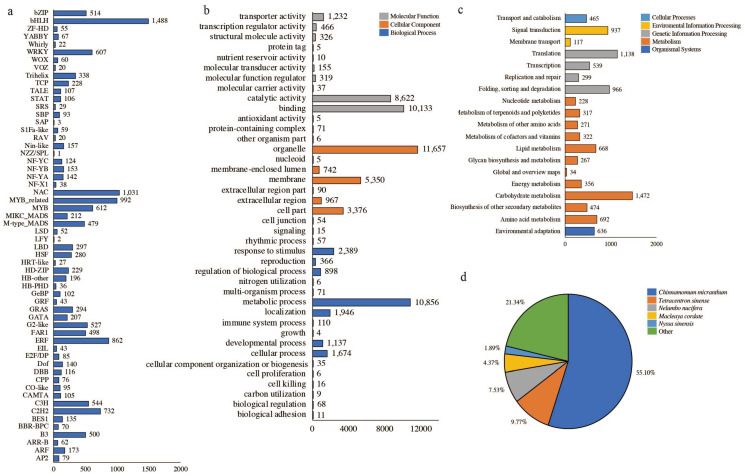
Gene function annotation information: (**a**) TF annotation information; (**b**) GO annotation information; (**c**) KEGG annotation information; (**d**) NR annotation information.

The detected genes were annotated using public databases, and the number of genes annotated to KEGG, GO, NR, NT, SwissProt, Pfam, KOG, and TF were 10,198 (33.76%), 26,652 (88.23%), 28,708 (95.03%), 22,256 (73.67%), 22,659 (75.01%), 8387 (27.76%), 18,191 (60.22%), and 14,364 (47.55%), respectively. The classification results of KEGG, GO, and TF are shown in Figure 4; of these, 914 (3.03%) genes were simultaneously annotated to eight databases, and 5, 6, and 5 genes were individually annotated to the TF, KEGG, and GO databases, respectively. By employing cluster analysis based on the expression pattern of the genes, the genes were grouped into 12 categories (Figure S1), of which the genes in categories 3, 6, and 8 were positively correlated with the phenotype of male sterility, with the number of genes being 8837 (29.25%). Additionally, 4883 (16.16%) genes in categories 9, 10, and 12 were negatively correlated with the male sterility phenotype (Figure S1).

### 2.3. Analysis of Differentially Expressed Genes

**Figure 5:**
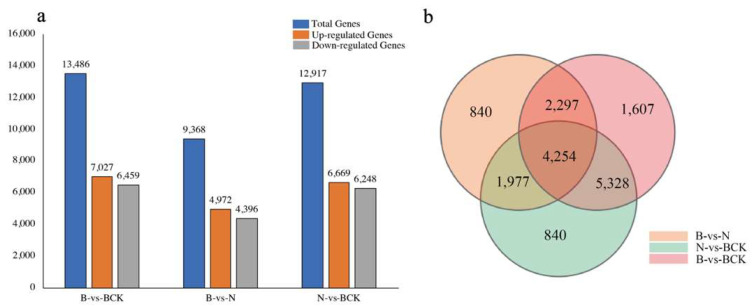
Differential gene overview: (**a**) differences between up- and downregulated genes in differential genes between groups; (**b**) differential gene duplication among different groups.

The comparative analysis of two-by-two differences revealed 17,661 differently expressed genes, with 13,486 (76.36%) differently expressed genes being the highest in the B and BCK groups, followed by 6669 (73.14%) in the N and BCK groups; the B and N groups presented the lowest number of differently expressed genes (4972, 53.04%) (Figure 5a). Among them, 1607, 1358, and 840 genes showed differences only in groups B vs. BCK, N vs. BCK, and B vs. N. Furthermore, two-by-two comparisons revealed that 4254 genes were differentially expressed in the three groups (Figure 5b). The clustering analysis of the expression patterns for 4254 differentially expressed genes showed that there were fewer genes in clusters 3, 6, and 8 compared to clusters 9, 10, and 12. Specifically, clusters 9, 10, and 12 had a total of 1366 genes, while clusters 3, 6, and 8 had a total of 902 genes.

**Figure 6:**
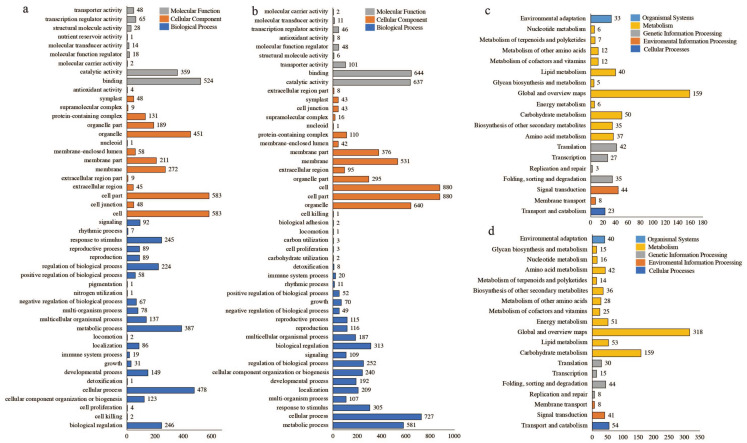
Information on positively and negatively related differently expressed genes using KEGG, GO classification: (**a**) GO classification for positively correlated differently expressed genes; (**b**) GO classification for negatively correlated differently expressed genes; (**c**) KEGG classification for positively correlated differently expressed genes; (**d**) KEGG classification for negatively correlated differently expressed genes.

The 2268 differentially expressed genes were categorized into positively and negatively correlated genes for KEGG and GO classification and enrichment analyses. The results showed significant differences between positively and negatively related genes across various categories, including the number of genes in different categories. The GO classification results showed that the gene types could be categorized into three main categories groups, with the same classification results in the cellular component group. One of the positively correlated genes in the biological process group belonged to nitrogen utilization and pigmentation. Furthermore, two negatively correlated genes belonged to biological adhesion and carbohydrate utilization each, and three belonged to carbon utilization and cell proliferation each; the remaining 22 categories were identical. One of the positively correlated genes in the molecular function group belonged to nutrient reservoir activity, and the remaining categories were the same as those observed in the negatively correlated categorization (Figure 6a,b). KEGG classification showed that both categories of genes could be classified into 5 major categories and 19 subcategories, among which the categories with the highest number of genes were global and overview maps, followed by carbohydrate metabolism; the third-highest number of genes among the positively correlated genes was for signal transduction, and the third-highest number of genes among the negatively correlated genes was for transport and catabolism (Figure 6c,d).

**Figure 7:**
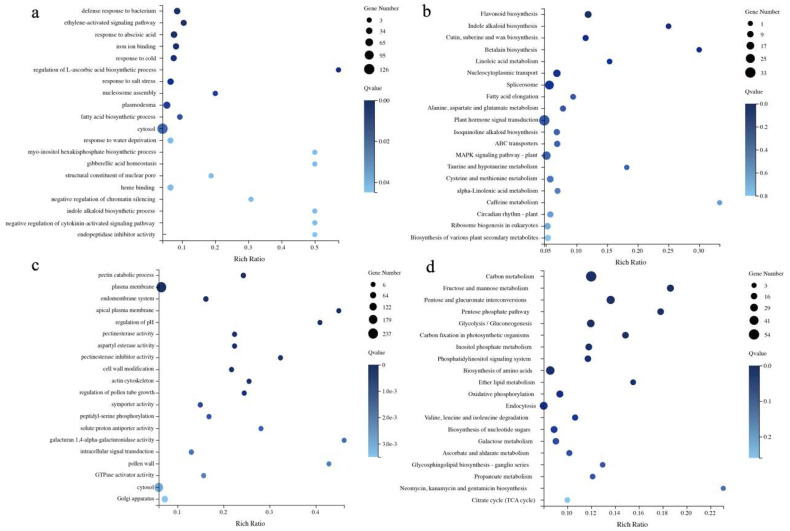
KEGG and GO enrichment information for positively and negatively correlated differently expressed genes: (**a**) GO enrichment for positively correlated differently expressed genes; (**b**) KEGG enrichment for positively correlated differently expressed genes; (**c**) GO enrichment for negatively correlated differently expressed genes; (**d**) KEGG enrichment for negatively correlated differently expressed genes.

The GO enrichment showed that the first three categories of positively related genes were GO:0042742 (defense response to bacteria), GO:0009873 (ethylene-activated signaling pathway), and GO:0009737 (response to abscisic acid), which were completely different from the negatively correlated gene enrichment results. The first three categories were GO:0045490 (pectin catabolic process), GO:0005886 (plasma membrane), and GO:0012505 (endomembrane system) (Figure 7a,c). KEGG enrichment analysis showed that the top three positively related genes were ko00941 (flavonoid biosynthesis), ko00901 (indole alkaloid biosynthesis), and ko00073 (cutin, suberin, and wax biosynthesis). The ko04075 (plant hormone signal transduction) genes had the highest number of genes, and the top three negatively related genes were ko01200 (carbon metabolism), ko00051 (fructose and mannose metabolism), and ko00040 (pentose and glucuronate interconversion) (Figure 7b,d).

### 2.4. DEG Analysis of the Starch and Sucrose Metabolism Pathway

**Figure 8:**
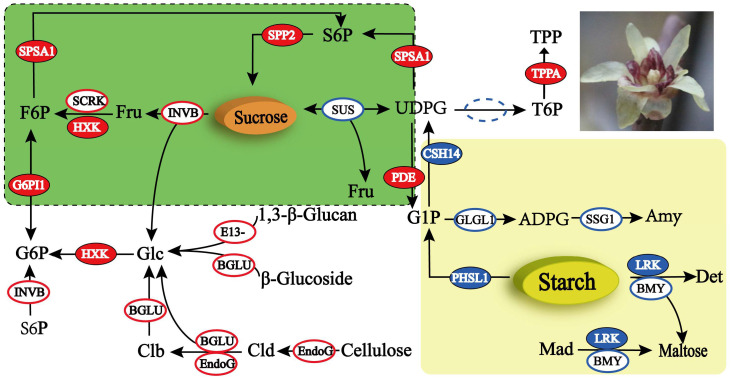
Starch and sucrose metabolism in male-sterile *C. praecox* material. Blue solid circles indicate the downregulation of the corresponding genes; blue hollow circles indicate the general downregulation of the corresponding genes; red solid circles indicate the upregulation of the corresponding genes, and red hollow circles indicate the general upregulation of the corresponding genes.

Screening was performed for genes related to the ko00500 (starch and sucrose metabolism) pathway from 2268 genes. A total of 45 DEGs were identified (Table S5), comprising 17 downregulated and 28 upregulated genes. In total, 18 enzymes were annotated, with the highest number of genes annotated to β-D-glucosidase (EC 3.2.1.21) with eight genes, followed by endo-1,4-β-D-glucanohydrolase (EC 3.2.1.4) with five genes combined, and invertase sucrase (EC 3.2.1.26) and β-1, 3-glucanase (EC 3.2.1.39) were annotated to four genes each (Figure 8). The functional analysis of the 45 DEGs revealed that the genes related to starch metabolism were more highly expressed during anther abortion. During the conversion of maltodextrin to maltose, three alpha-amylase genes (EC 3.2.1.1) were expressed at higher levels in group B than in groups N and BCK. During the conversion of starch to UDPG, the phosphorylase (EC 2.4.1.1) and UDP–glucose pyrophosphorylase (EC 2.7.7.9) genes of the two classes of enzymes were differentially expressed and were the highest in group B. During the conversion of starch to dextrin and amylose, six genes belonging to three classes of enzymes were screened; the three genes that were downregulated had the highest expression in group B, and the three genes that were upregulated had the highest expression in the BCK group (Figure 8). The genes related to sucrose and cellulose metabolism were highly expressed during normal anther development. Four genes belonged to invertase (EC 3.2.1.26) in the conversion of sucrose to d-fructose and d-glucose, two of which were downregulated with the highest expression of >100, and the remaining two were upregulated, with expression below 10. Thirteen genes from two classes of enzymes were screened for the conversion of cellulose to d-glucose, of which nine genes were upregulated, and their expression was significantly lower in group B than in groups N and BCK. A total of 12 genes from 6 classes of enzymes were screened for d-fructose and d-glucose metabolism, of which 9 genes were upregulated, and the remaining 3 genes were expressed below 20 (Figure S2 and Figure 8).

### 2.5. DEG Analysis of the Plant Hormone Signal Transduction Pathway

**Figure 9:**
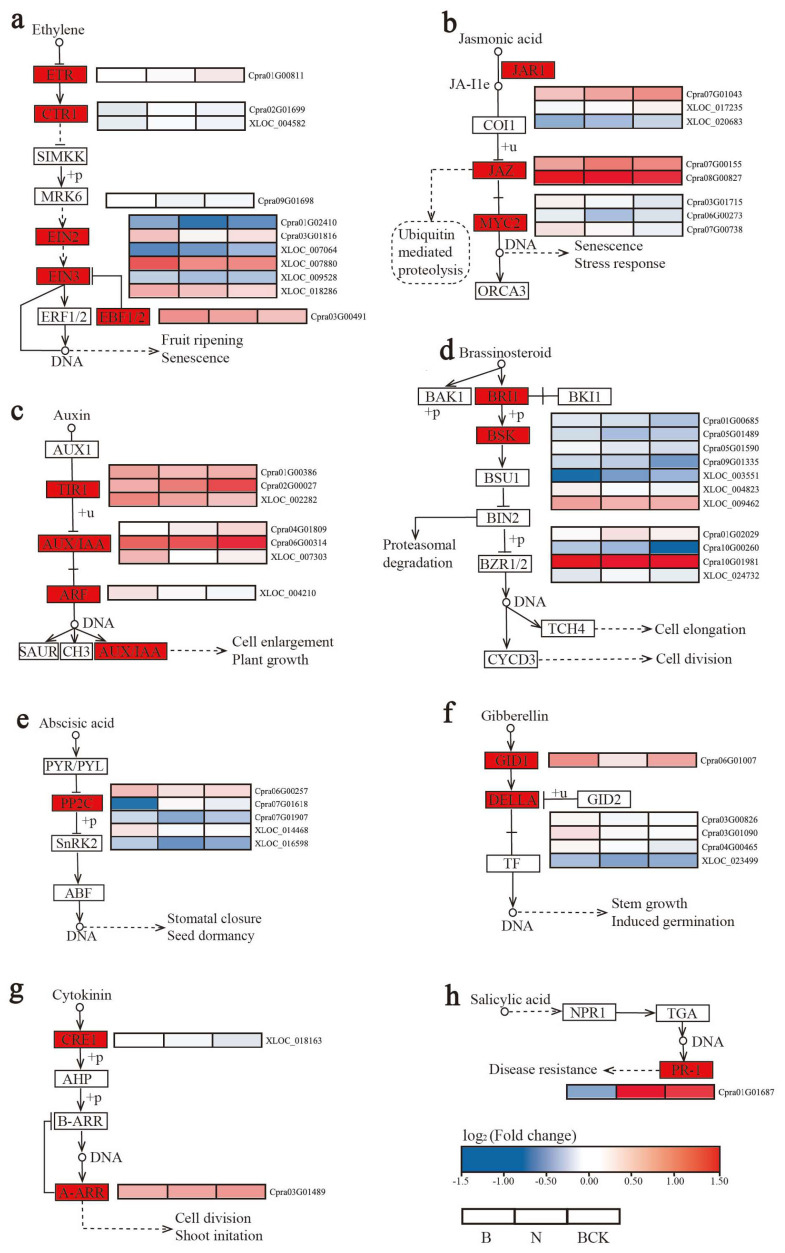
Differentially expressed genes in the hormone signal transduction pathway (ko04075) in male-sterile *C. praecox* material: (**a**) ethylene signal transduction pathway; (**b**) jasmonic acid signal transduction pathway; (**c**) auxin signal transduction pathway; (**d**) brassinosteroid signal transduction pathway; (**e**) abscisic acid signal transduction pathway; (**f**) gibberellin signal transduction pathway; (**g**) cytokinin signal transduction pathway; (**h**) salicylic acid signal transduction pathway.

Screening was performed for ko04075 (plant hormone signal transduction) pathway-related genes from 2268 genes. DEGs were screened in the signal transduction processes of all 8 classes of plant endogenous hormones, totaling 50 DEGs (33 upregulated genes and 17 downregulated genes) belonging to 20 classes of regulatory factors (Figure 9 and Table S6). The ethylene signal transduction pathway was screened for the most regulators, with five containing eleven DEGs, followed by jasmonic acid and auxin, both of which included three regulators with eight and seven DEGs, respectively. Among the five regulators of the ethylene signal transduction pathway, EIN2 (1) and EBF1/2 (1) were downregulated; five regulators of EIN3 (6) were upregulated, one was downregulated, and ETR (1) and CTR1 (2) were upregulated. The jasmonic acid signal transduction pathway was downregulated with MYC2 (3), upregulated with JAR1 (3), and downregulated with one gene in JAZ (2). In the auxin signal transduction pathway, two genes in TIR1 (3) and ARF (1) and one gene in AUX/IAA (3) were downregulated. The gibberellin signal transduction pathway was downregulated with class 2 regulators GID1 (1) and DELLA (4). Two classes of regulators, totaling 11 genes, were screened in the brassinosteroid signal transduction pathway, with eight downregulated genes and three upregulated genes; two genes were downregulated in BSK (4), and six were downregulated in BRI1 (7). Furthermore, the abscisic and salicylic acid signal transduction pathways were screened for the differential expression of only one class of regulator, with four genes downregulated with PP2C (5) in the abscisic acid signal transduction pathway and upregulated with PR-1 (1) in the salicylic acid signal transduction pathway. The cytokinin signal transduction pathway was screened for two classes of regulators, with CRE1 (1) downregulated and A-ARR (1) upregulated.

### 2.6. Verification of DEGs by qPCR

**Figure 10:**
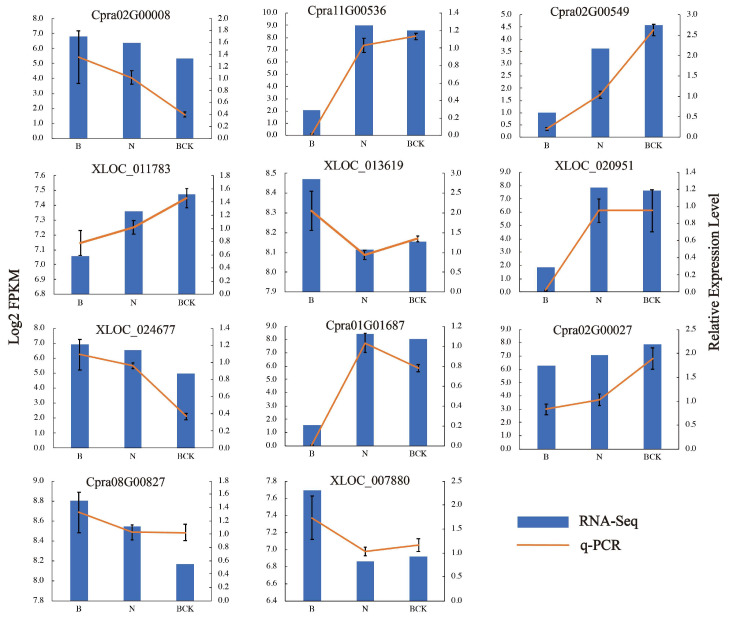
Comparison of qPCR results with RNA-Seq results.

To validate the transcriptome data, we selected 11 DEGs for confirmation by qPCR. These included seven genes (*Cpra02G00008/EndoG*, *Cpra11G00536/EndoG*, *Cpra02G00549/ BGLU*, *XLOC_011783/SPP2*, *XLOC_013619/SUS*, *XLOC_020951/E13B*, and *XLOC_024677/E1313*) in the starch and sucrose metabolism pathways and four genes (*Cpra01G01687/PR-1*, *Cpra02G00027/TIR1*, *Cpra08G00827/JAZ*, and *XLOC_007880/EIN3*) in the plant hormone signal transduction pathway. The qPCR results were consistent with the transcriptome data (Figure 10).

## 3. Discussion

As a traditional winter ornamental and economic flowering tree endemic to China, *C. praecox* has a long history of cultivation and a rich variety of species and types [4,21]. Furthermore, *C. praecox* is a heterogamous pollinated plant that is easy to hybridize in nature; therefore, it exhibits extensive trait variation in its progeny [22]. The mating systems of plants are complex and diverse and can be categorized into seven types, with a high degree of variability among different plant mating systems. This typically includes various reproductive strategies, such as selfing, outcrossing, and parthenogenetic selfing (mixed mating) [23,24]. *C. praecox* produces hermaphroditic flowers and mainly experiences heterosis, with limited self-fertilization, which is largely prevented through dichogamy and herkogamy [18]. Dichogamy, also known as floral disjunction, refers to the temporal separation of male and female functions of hermaphroditic plants, which prevents the interference of male and female functions, promotes heterogamy, and improves the fitness of the population [17]. However, regardless of temporal or spatial segregation, *C. praecox* still participates in self-pollination, which results in self-fertilization [18].

Male sterility, a genetic phenomenon that refers to the inability to produce normal anthers, pollen, or male gametes during sexual reproduction, is common in many flowering plants [25]. Cytoplasmic male sterility has been observed in >200 species [26]; however, no study has been conducted on male sterility in *C. praecox*. During a survey of *C. praecox* species resources, our team discovered pollen-abortive *C. praecox* species (CP-MS). Observations of CP-MS phenotypes revealed that pollen was primarily abortive from the bud stage to the full blooming stage, indicating that anthers were abortive before pistil maturity and that the probability of self-pollination in CP-MS was significantly reduced. During the blooming period of CP-MS, the tepals rolled backward, exposing the pistil and stamen completely, and the inner tepals were brightly colored. Regarding plants that primarily undergo insect pollination, flower color and corolla structure directly affect pollination efficiency [27]. Notably, the structure and color of CP-MS flowers increase the chance of heterogamous pollination. Male-sterile material in *C. praecox* is crucial for heterogamous mating; it enables the elimination of the tedious process of male organ removal in breeding, simplifies the procedure, and significantly reduces the consumption of nutrients. Male sterility is an important tool for the genetic improvement in plants and the optimization of production using hybrid advantages and is a classic model for exploring cytoplasmic mechanisms and reversing signaling pathways in anther and pollen developmental biology [28].

The primary modes for the inheritance of male sterility in plants are cytoplasmic male sterility (CMS) and nuclear sterility (GMS). The inheritance of nuclear male sterility generally conforms to typical Mendelian inheritance patterns; however, cytoplasmic male sterility is inherited in a matrilineal fashion and is controlled by the nucleus and the cytoplasm [29]. Phenotypes of cytoplasmic male sterility are primarily associated with pollen, anther, and stamen development. Pollen abortion is a common type of cytoplasmic sterility; it is mainly characterized by gametophyte abortion. Cytoplasmic male sterility is a biological phenomenon caused by mutation, recombination, or rearrangement of the mitochondrial genome and has been identified in >200 species [26,30]. Pollen development is crucial in the life cycle of seed plants and plays an essential role in plant seed formation and the development of fruits [31]. Pollen development begins with the pollen mother cell (PMC) undergoing mitosis and meiosis to produce tetrads and subsequently transitioning into the mononucleate, binucleate, and trinucleate phases to finally develop into mature pollen grains. Any blockage or developmental abnormality in this process can inhibit functional pollen grain formation, resulting in male sterility [32]. Anther abortion in plants is primarily associated with abnormalities in the pollen wall, intermediate cells, and tapetal cells, and it modulates structural abnormalities in the filament and anther diaphragm vascular bundle [33]. The period and manner of microspore abortion in different sterile male lines are not comparatively consistent [34]. There are three types of male-sterile lines in kale-type *Brassica napus*: no pollen sac type, no PMC type, and no meiotic process [35]. Male sterility in *Lycium barbarum* begins abnormally at the tetrad stage, and callus wall degradation does not occur, inhibiting the production of fertile pollen grains [36]. Amphidiploid microspore abortion in *Nyssa yunnanensis* is due to the abnormal development of tapetal cells during the tetrad and the single microspore stages [37]. Morphological observations of CP-MS led us to hypothesize that male sterility in CP-MS may occur during the premature stage of pollen grain maturation.

The development of anthers is a tightly regulated biological process, and several key genes play regulatory roles in the development of anthers. Mutations or differential expression of these genes may lead to male sterility [38]. Male sterility is important for breeding, and its combination with transcriptomic technology is crucial for exploring the molecular mechanisms underlying male sterility. Transcriptome sequencing technology, which emerged in 2008, can be employed to construct gene regulatory networks and screen key genes by assessing transcriptomes in different tissues from different subjects and different developmental periods [39]. It has been extensively used in evaluating the molecular mechanism of male sterility in *Zea mays* [40], *Brassica campestris* [38], *Chrysanthemum morifolium* [41], and *Lycium barbarum* [42]. In this study, we used transcriptome sequencing technology to analyze the differences between the aborted anthers of CP-MS varieties and the normal anther development of *C. praecox* varieties. A total of 4254 DEGs were screened; GO and KEGG classification and enrichment analyses indicated that the anther abortion of *C. praecox* may be related to the starch and sucrose metabolism pathways and hormone signal transduction pathways. Abnormalities in mitochondrial energy synthesis, phytohormone signaling, and starch and sucrose metabolism have been found in sterile male lines of *Hevea brasiliensis* [43]. Studies on anther abortion in *Lycium barbarum* have revealed that DEGs are primarily enriched in pathways related to amino acid biosynthesis, phytohormone signaling, phenylpropanoid biosynthesis, starch and sucrose metabolism, fatty acid metabolism, and other related pathways [44]. Transcriptomic studies on male sterility in *Zea mays* and *Medicago sativa* have shown that DGEs are primarily enriched in pathways such as sucrose and starch metabolism, synthesis of the glycolytic pentose phosphate pathway, and hormone signaling [45,46].

Carbohydrate metabolism is a basic metabolic pathway for plant growth and development. Sugar provides energy and nutrients for growth and development, and carbohydrates are one of the main components of the cell wall. Disturbances in sugar metabolism can lead to abnormal pollen development [47]. Studies on the cytoplasmic male-sterile line of soybean revealed that the soluble sugar and soluble protein contents in the bracts were lower than those in the homozygous maintained line [48]. Proteins involved in carbohydrate and energy metabolism differed in sterile lines in a study conducted on *Brassica napus* [49]. The comparative analysis of the anther-aborting materials and normal anther-developing materials of *C. praecox* revealed that the gene expression of enzymes in the starch degradation pathway of the anther-aborting materials was higher than that of the normal anther-developing materials during the same period. In contrast, the gene expression of enzymes involved in the cellulose and sucrose degradation pathways was mostly lower than that in materials with normal anther development. The energy expenditure of anther abortion in *C. praecox* at the budding period was significantly different from that of plants with normal anther development. Starch and sucrose metabolism provide nutrients for microspore development, and a lack of sugar and starch may cause metabolic disorders that affect microspore formation, leading to male sterility [45].

Notably, regarding cytoplasmic male sterility, there were significant differences in endogenous hormones between sterile and maintained lines [50]. Similar results have been reported for *Beta vulgaris*, *Capsicum annuum*, *Brassica napus*, *Linum usitatissimum*, and *Oryza sativa* [51,52,53,54,55]. In this study, we found that the gene expression of several plant hormones during signal transduction was generally higher in *C. praecox* anther aborting variety than in normally developing varieties by analyzing the differences between anther aborting and normally developing materials. Among them, the ethylene signal transduction pathway had the most differentially expressed genes, followed by jasmonic acid and auxin; additionally, GO and KEGG enrichment analyses of the differentially expressed genes revealed that they might be related to ethylene synthesis. The overexpression of the ethylene synthesis gene *CsACO2* inhibits the development of stamens of *Arabidopsis thaliana* [56]. Furthermore, the stamen deletion and deformity exhibited by *Nicotiana tabacum* may be associated with endogenous ethylene [30]. Jasmonic acid regulates pollen maturation and anther dehiscence in plants. The blockage of jasmonic acid synthesis inhibits anther dehiscence, leading to pollen abortion [57]. Notably, auxin is a key regulator of late developmental processes (anther dehiscence, pollen maturation, and pre-anthesis filament elongation) during the maturation of *Arabidopsis thaliana* stamen [58]. It has been hypothesized that anther abortion in *C. praecox* is closely related to ethylene, jasmonic acid, and auxin. Based on these results, it is tentatively speculated that male sterility in *C. praecox* may be caused by abnormalities in certain metabolic processes under the influence of different hormones. The observed differences unveil a more complex phenomenon that could be further assessed using the large genetic diversity of available genotypes.

## 4. Materials and Methods

### 4.1. Plant Material

The male-sterile (CP-MS) and normally developing plant materials (CP-N) of *C. praecox* were cultivated in the germplasm nursery of the College of Horticulture and Landscape Architecture, Southwest University of China. Detailed information on the names and geographic origins is presented in Table S7. The flower buds of the corresponding varieties were collected at the full-bloom stage in December 2023. The tepals were carefully peeled off to retain the ovaries, pistils, and stamens. They were then placed into 1.5 mL RNase-free tubes. Twelve samples were collected in each centrifuge tube, and the procedure was repeated three times. The samples were treated with liquid nitrogen and stored in an ultra-low-temperature refrigerator at −80 °C in the floriculture laboratory of Southwest University before RNA extraction and transcriptome sequencing.

### 4.2. Paraffin Sections

CP-MS and CP-N varieties were sampled, with 20 specimens each, preserved in FAA fixative and stored at 4 °C. Specimens underwent dehydration in a series of ethanol concentrations (30%, 50%, 70%, 85%, 95%, and 100%), each step lasting 1 h. Post-dehydration, samples were immersed in a solution of 100% ethanol and xylene for 1 h, followed by pure xylene for 1 h for transparency. Subsequently, specimens were incubated in high-melting-point paraffin at 60–62 °C for 3 h. Samples were then embedded in paraffin, sectioned into 7–9 μm slices, affixed to slides, deparaffinized in xylene, rehydrated in graded ethanol series, and stained with eosin and fast green for enhanced contrast. Imaging and analysis were performed using an advanced stereomicroscope (Nikon SMZ1000, Minato, Japan).

### 4.3. RNA Extraction, Library Construction, and Sequencing

The total RNA was extracted using an RNArep Pure kit (Beijing Tiangen Biotechnology Co., Ltd., Beijing, China). The quality of the RNA was determined using 1% agarose gel electrophoresis and quantified using a NanoDrop ND-1000 Spectrophotometer (Thermo Fisher Scientific, Wilmington, MA, USA). The library was constructed by enriching mRNA using oligo (dT) magnetic beads after fragmentation. Reverse transcriptase was used to synthesize a first-strand cDNA, which then served as a template to synthesize a double-stranded cDNA for end repair. Next, a single ‘A’ nucleotide was added to the 3′ end, and an aptamer was set up and attached to the cDNA. The quality of library construction was checked by amplifying the final library using phi29 DNA polymerase. DNA nanoballs (DNBs) containing the library were loaded into patterned nanoarrays, and 100 base reads were generated by sequencing the terminals on the BGIseq500 platform (BGIseq500, Shenzhen, China). Measurements were performed in triplicate.

### 4.4. Assembly, Functional Annotation, and DEG Analysis

Raw data were filtered using SOAPnuke (v1.5.2) [59], removing reads with aptamers; reads with an unknown base ratios > 10%, reads with a low-quality base ratios > 50%, and clean reads were saved in FASTA format. After obtaining clean reads and downloading genome data of *C. praecox* [60] from published databases, we used HISAT to align them with the reference genome sequence. Functional databases, namely Kyoto Encyclopedia of Genes and Genomes (KEGG), Gene Ontology (GO), National Center for Biotechnology Information Nonredundant Protein Sequences (NR), Nucleotide Sequence Data Bank (NT), Swiss-Prot Sequence Data Bank (SwissProt), Protein Family Data Bank (Pfam), and Nuclear Homologous Proteome of Europe (KOG), were employed to annotate single genes and predict transcription factors. The gene expression levels were calculated by RSEM (v1.3.1) [61]. The sequence analysis software used was Mfuzz-2.6.0, which is based on a loose clustering algorithm. Mfuzz is an R-based package that clusters genes based on similar expression profiles, helping to find functionally similar genes [62]. The heatmap was drawn by pheatmap (v1.0.8) [63] according to the gene expression difference in the different samples. Essentially, differential expression analysis was performed using the DESeq2(v1.4.5) [64]. To gain insight into phenotypic changes, GO (http://www.geneontology.org/) and KEGG (https://www.kegg.jp/) enrichment analysis of annotated differentially expressed genes was performed by Phyper based on hypergeometric tests. The significant levels of terms and pathways were corrected by Q value with a rigorous threshold (Q value ≤ 0.05) [65].

### 4.5. Validation of DEGs Using Quantitative PCR (qPCR)

Using the extracted RNA, cDNA was reverse-transcribed according to the instructions of the All-in-One First-Strand Synthesis MasterMix (dsDNase) kit (Jiangsu Yugong Biotechnology Co., Ltd., Lianyungang, China). Three biological replicates and three technical replicates were performed independently using the 2×TSINGKE^®^ Master qPCR Mix (SYBR Green I) Kit (Beijing Kengke Biotechnology Co., Ltd., Beijing, China), referring to the instructions for qPCR. The qPCR analyses were performed by Bio-RAD CFX96 (Bio-RAD CFX Manager Software Version 1.6). The primer sequences are shown in Table S8. *Cp18s* and *CpRPL8* were selected as the reference genes [66]. The relative expression levels of the target genes were calculated using the 2^−ΔΔCT^ method [67].

## 5. Conclusions

Our results suggest that male sterility in *C. praecox* CP-MS occurs before pollen grain maturity. Transcriptome analysis revealed a large number of DEGs between the CP-MS of anther-aborting and normal anther-developing varieties. The differentially expressed genes were significantly enriched in carbon metabolism, flavonoid biosynthesis, pectin catabolic processes, and defense responses to bacteria. Furthermore, the expression of genes related to the CP-MS starch degradation pathway was high; however, the expression of genes related to the cellulose and sucrose degradation pathways was low. The expression of genes related to phytohormone signaling, such as ethylene, jasmonic acid, and auxin, was high. Based on these results, it is tentatively speculated that male sterility in *C. praecox* may be caused by abnormalities in certain metabolic processes under the influence of different hormones. In summary, our results reveal transcriptomic differences between the two stamen types and provide insights into important metabolic pathways that may influence male sterility in *C. praecox*.

## Data Availability

The clean data of RNA-seq generated in this study have been submitted to the BioProject database of the National Center for Biotechnology Information, number PRJNA1092740.

## Supplementary Materials

The following supporting information can be downloaded at: https://www.mdpi.com/article/10.3390/plants13182571/s1, Figure S1. Results of classification of expression patterns of genes. Figure S2. Heatmap of differential gene expression in starch and sucrose metabolism pathways (ko00500). Table S1. Clean reads quality statistics. Table S2. Comparative analysis of reference genomes. Table S3. Reference gene sequence alignment. Table S4. Transcript length distribution of known genes. Table S5. The expression levels of genes in the starch and sucrose metabolism pathway of *C. praecox* (FPKM). Table S6. The expression levels of genes in the plant hormone signal transduction pathway of *C. praecox* (FPKM). Table S7. Information on plant materials. Table S8. Information on qPCR primer sequences.

## References

[1] Yang J., Dai P., Zhou T., Huang Z., Feng L., Su H., Liu Z., Zhao G. (2013). Genetic diversity and structure of wintersweet (*Chimonanthus praecox*) revealed by EST-SSR markers. Sci. Hortic..

[2] Shang J., Tian J., Cheng H., Yan Q., Chen L. (2020). The chromosome-level wintersweet (*Chimonanthus praecox*) genome provides insights into floral scent biosynthesis and flowering in winter. Genome Biol..

[3] Sui S., Luo J., Ma J., Zhu Q., Lei X., Li M. (2012). Generation and analysis of expressed sequence tags from *Chimonanthus praecox* (Wintersweet) flowers for discovering stress-responsive and floral development-related genes. Int. J. Genom..

[4] Chen L. (2012). Research advances on Calycanthaceae. Chin. Landsc. Archit..

[5] Kitagawa N., Ninomiya K., Okugawa S., Motai C., Nakanishi Y., Yoshikawa M., Muraoka O., Morikawa T. (2016). Quantitative determination of principal alkaloid and flavonoid constituents in wintersweet, the flower buds of *Chimonanthus praecox*. Nat. Prod. Commun..

[6] Wang Z., Zhang N., Ma X., Yang J., Li X., Pang W., Lin C. (2019). Chemical composition and in vitro antibacterial and antifungal activity of essential oils from different parts of *Chimonanthus praecox* (Linn.) Link. Chin. J. Mod. Appl. Pharm..

[7] Chen L., Lu D. (2001). Cultivar classification system of *Chimonanthus praecox* (L.) Link. J. Beijing For. Univ..

[8] Hedrick P.W. (2004). Recent developments in conservation genetics. For. Ecol. Manag..

[9] Li F. (2017). Analysis of Genetic Diversity and Mating System in Natural Populations of *Ormosia hosiei*. Ph.D. Thesis.

[10] Audigeos D., Brousseau L., Traissac S., Scotti-Saintagne C., Scotti I. (2013). Molecular divergence in tropical tree populations occupying environmental mosaics. J. Evol. Biol..

[11] Berg H. (2003). Factors influencing seed: Ovule ratios and reproductive success in four cleistogamous species: A comparison between two flower types. Plant Biol..

[12] Zhang X., Wang G., Zhang S., Chen S., Wang Y., Wen P., Ma X., Shi Y., Qi R., Yang Y. (2020). Genomes of the banyan tree and pollinator wasp provide insights into fig-wasp coevolution. Cell.

[13] Wang R., Jia H., Wang J., Zhang Z. (2010). Flowering and pollination patterns of Magnolia denudata with emphasis on anatomical changes in ovule and seed development. Flora-Morphol. Distrib. Funct. Ecol. Plants.

[14] Wang Q., Chen S., Chen L., Liu B., Li H. (2018). Research advances on dichogamy of Walnut. China Fruits.

[15] Li L., Lu N., Fan B., Zhao Z. (2016). Effect of flowering time on floral sexual durations and phenotypic gender in dichogamous Aconitum gymnandrum. Biodivers. Sci..

[16] Jin X., Xiao C., Zhang J., Zhao J., Xiao Z., Zhang H., Jin Z. (2021). Comparison study of flowering process in three dichogamous Lauraceae species. J. Trop. Subtrop. Bot..

[17] Lloyd D.G., Webb C. (1986). The avoidance of interference between the presentation of pollen and stigmas in angiosperms I. Dichogamy. N. Z. J. Bot..

[18] Lv C., Liu L., Zhang L., Hu Q., Chen M. (2017). Characteristics of pollination ecology for wintersweet (*Chimonanthus praecox* ( L. ) Link) after transplanted to Yantai. J. Ludong Univ. (Nat. Sci. Ed.).

[19] Worboys S.J., Jackes B.R. (2005). Pollination processes in Idiospermum australiense (Calycanthaceae), an arborescent basal angiosperm of Australia’s tropical rain forests. Plant Syst. Evol..

[20] Li C. (2023). Revealing the Genetic Basis of Male Sterility Based on the Evolution of Citrus Cytonuclear. Ph.D. Thesis.

[21] Shen Z., Li W., Li Y., Liu M., Cao H., Provart N., Ding X., Sun M., Tang Z., Yue C. (2021). The red flower wintersweet genome provides insights into the evolution of magnoliids and the molecular mechanism for tepal color development. Plant J..

[22] Zhao B., Zhang Q. (2007). Genetic diversity of germplasm resources of *Chimonanthus praecox* (L.) Link based on AFLP marker. Acta Ecol. Sin..

[23] Baránková S., Pascual-Díaz J.P., Sultana N., Alonso-Lifante M.P., Balant M., Barros K., D’Ambrosio U., Malinská H., Peska V., Lorenzo I.P. (2020). Sex-chrom, a database on plant sex chromosomes. New Phytol..

[24] Li L., Wang Q., Xiao Y., Chen X., Chen X., Hu X. (2023). On the theories of plant mating system and molecular evolution and their applications. Sci. Sin. (Vitae).

[25] Singh S., Dey S., Bhatia R., Kumar R., Behera T. (2019). Current understanding of male sterility systems in vegetable Brassicas and their exploitation in hybrid breeding. Plant Reprod..

[26] Hu J., Huang W., Huang Q., Qin X., Yu C., Wang L., Li S., Zhu R., Zhu Y. (2014). Mitochondria and cytoplasmic male sterility in plants. Mitochondrion.

[27] Ramsey M. (1995). Ant pollination of the perennial herb Blandfordia grandiflora (Liliaceae). Oikos.

[28] Zhang Z., Zhao H., Hu M., Den L., Wang Q., Li J., Yuan H. (2019). Application progress of Omics in the research of anther development I: Transcriptomics. Curr. Biotechnol..

[29] Vedel F., Pla M., Vitart V., Gutierres S., Chétrit P., Paepe R.d. (1994). Molecular basis of nuclear and cytoplasmic male sterility in higher plants. Plant Physiol. Biochem..

[30] Chen H., Qian B., Pei X., Li Y., Huang C., Liu Y., Yuan C., Yi H., Zeng J., Yi B. (2023). Cytological and transcriptome analysis of cytoplasmic male sterility and maintainer line in glu-CMS tobacco. Acta Tabacaria Sin..

[31] Herbert S.W., Walton D.A., Wallace H.M. (2019). Pollen-parent affects fruit, nut and kernel development of Macadamia. Sci. Hortic..

[32] Chase C.D. (2007). Cytoplasmic male sterility: A window to the world of plant mitochondrial–nuclear interactions. Trends Genet..

[33] Wei B. (2017). Inheritance and Candidate Genes Identification for the Fertility Restorer of Cytoplasmic Male Sterility in Pepper. Ph.D. Thesis.

[34] He C., Liu Z., Xiong X., Zou X., Xiao L. (2008). Cytologic observations on anther development of 9704A, a cytoplasmic male sterile line in *Capsicum annum* L. Acta Hortic. Sin..

[35] Nie M., Wang G., Zhu W. (2007). Cytology research on the anther abortion of three male sterility lines in Rapeseed (*Brassica napus* L.). Sci. Agric. Sin..

[36] Zhou T., Wei Y., Liang W., Guan C., Yang S. (2023). Transcriptome analysis of gene expression characteristics at anther development stages of the male sterile in Wolfberry (*Lycium barbarum*). Genom. Appl. Biol..

[37] Kang H., Zhang S., Luo T., Chen J., Yang W. (2019). Development mechanism of anther on hermaphrodite flower in Nyssa yunnanensis. J. West China For. Sci..

[38] Wang J., Tang X., Yuan L., Chen G., Hou J., Yang Y., Huang X., Wang C. (2023). Integrated analysis of transcriptome and proteome changes related to the male-sterile mutant MS7-2 in wucai (*Brassica campestris* L.). Sci. Hortic..

[39] Wang Z., Gerstein M., Snyder M. (2009). RNA-Seq: A revolutionary tool for transcriptomics. Nat. Rev. Genet..

[40] Li C., Zhao Z., Liu Y., Liang B., Guan S., Lan H., Wang J., Lu Y., Cao M. (2017). Comparative transcriptome analysis of isonuclear-alloplasmic lines unmask key transcription factor genes and metabolic pathways involved in sterility of maize CMS-C. PeerJ.

[41] Li F., Chen S., Chen F., Teng N., Fang W., Zhang F., Deng Y. (2010). Anther wall development, microsporogenesis and microgametogenesis in male fertile and sterile Chrysanthemum (Chrysanthemum morifolium Ramat., Asteraceae). Sci. Hortic..

[42] Fan W., Liu X., Ma X., Yang H., Zhu J., Tang J., Yue S., Zheng R. (2023). Mining of male sterility-related genes in *Lycium barbarum* using transcriptome data. Chin. Tradit. Herb. Drugs.

[43] Fu T., Wang Y., Gao H., Zhuang N. (2023). Expression and bioinformatics analysis of male sterility-related gene HbBPC7 in Hevea brasiliensis. Mol. Plant Breed..

[44] Zhang X., Feng J., Chen J., Fan W., Wang L., Yue S., Zhen R. (2022). Comparative transcriptome analysis of male sterile anther in Wolfberry (*Lycium barbarum* L. ). Genom. Appl. Biol..

[45] Yan J., Wang J., Zhao Y., Jia X., Guo J., Zhu L. (2023). Transcriptome analysis of anthers of the maize CMS-S type sterile line. J. Hebei Agric. Univ..

[46] Yu W., Miao Y., Mu H., Jia X., Yan D., Xu B. (2023). Transcriptomic analysis on the mechanism of polen abortion in male sterile lines of Alfalfa. Acta Agrestia Sin..

[47] Kretschmer M., Croll D., Kronstad J.W. (2017). Maize susceptibility to Ustilago maydis is influenced by genetic and chemical perturbation of carbohydrate allocation. Mol. Plant Pathol..

[48] He D. (2020). Transcriptome Analysis of Cytoplasmic Male Sterile Lines and Their Maitainers in Soybean. Master’s Thesis.

[49] Heng S., Chen F., Wei C., Li X., Yi B., Ma C., Tu J., Shen J., Fu T., Wen J. (2019). Cytological and iTRAQ-based quantitative proteomic analyses of hau CMS in *Brassica napus* L. J. Proteom..

[50] Sawhney V.K., Shukla A. (1994). Male sterility in flowering plants: Are plant growth substances involved?. Am. J. Bot..

[51] Wang H., Wu Z., Han Y. (2008). Relationships between endogenous hormone contents and cytoplasmic male sterility in sugarbeet. Sci. Agric. Sin..

[52] Wu Z., Hu K., Fu J., Qiao A. (2010). Relationships between cytoplasmic male sterility and endogenous hormone content of pepper bud. J. South China Agric. Univ..

[53] Dubas E., Janowiak F., Krzewska M., Hura T., Żur I. (2013). Endogenous ABA concentration and cytoplasmic membrane fluidity in microspores of oilseed rape (*Brassica napus* L.) genotypes differing in responsiveness to androgenesis induction. Plant Cell Rep..

[54] Tianxia G., Zhanhai D., Jianping Z. (2007). Studies on the changes of phytohormones during bud development stage in thermo-sensitivity genic male-sterile flax. Chin. J. Oil Crop Sci..

[55] Huang S., Zhou X. (1994). Relationship between rice cytoplasmic male sterility and contents of GA_(1+ 4) and IAA. Acta Agric. Boreali-Sin..

[56] Duan Q., Wang D., Xu Z., Bai S. (2008). Stamen development in Arabidopsis is arrested by organ-specific overexpression of a cucumber ethylene synthesis gene CsACO_2_. Planta.

[57] Caldelari D., Wang G., Farmer E.E., Dong X. (2011). Arabidopsis lox3 lox4 double mutants are male sterile and defective in global proliferative arrest. Plant Mol. Biol..

[58] Cecchetti V., Altamura M.M., Falasca G., Costantino P., Cardarelli M. (2008). Auxin regulates Arabidopsis anther dehiscence, pollen maturation, and filament elongation. Plant Cell.

[59] Chen Y., Chen Y., Shi C., Huang Z., Zhang Y., Li S., Li Y., Ye J., Yu C., Li Z. (2018). SOAPnuke: A MapReduce acceleration-supported software for integrated quality control and preprocessing of high-throughput sequencing data. Gigascience.

[60] Jiang Y., Chen F., Song A., Zhao Y., Chen X., Gao Y., Wei G., Zhang W., Guan Y., Fu J. (2023). The genome assembly of *Chimonanthus praecox* var. concolor and comparative genomic analysis highlight the genetic basis underlying conserved and variable floral traits of wintersweet. Ind. Crops Prod..

[61] Li B., Dewey C.N. (2011). RSEM: Accurate transcript quantification from RNA-Seq data with or without a reference genome. BMC Bioinform..

[62] Kumar L., Futschik M.E. (2007). Mfuzz: A software package for soft clustering of microarray data. Bioinformation.

[63] Kolde R., Kolde M.R. (2015). Package ‘pheatmap’. R Package.

[64] Love M.I., Huber W., Anders S. (2014). Moderated estimation of fold change and dispersion for RNA-seq data with DESeq2. Genome Biol..

[65] John D.S., Andrew J.B., Alan D., David R., Gregory W. Qvalue: Q-Value Estimation for False Discovery Rate Control. R Package Version 2.26.0. http://github.com/jdstorey/qvalue.

[66] Chen S., Zhao W., Fu X., Guo C., Cai Y., Huang S., Chen L., Yang N. (2022). Screening and verification of reference genes of wintersweet (Chimonanthus praecox L.) in real-time quantitative PCR analysis. Mol. Plant Breed..

[67] Livak K.J., Schmittgen T.D. (2001). Analysis of relative gene expression data using real-time quantitative PCR and the 2^−ΔΔCT^ method. Methods.

