# Supplementary figures and images for: Morphological and Transcriptomic Analyses Reveal the Involvement of Key Metabolic Pathways in Male Sterility in Chimonanthus praecox (L.) Genotypes
Source: Plants (Basel). 2024 Sep 13;13(18):2571. doi: 10.3390/plants13182571 (PMC11435207; doi:10.3390/plants13182571)

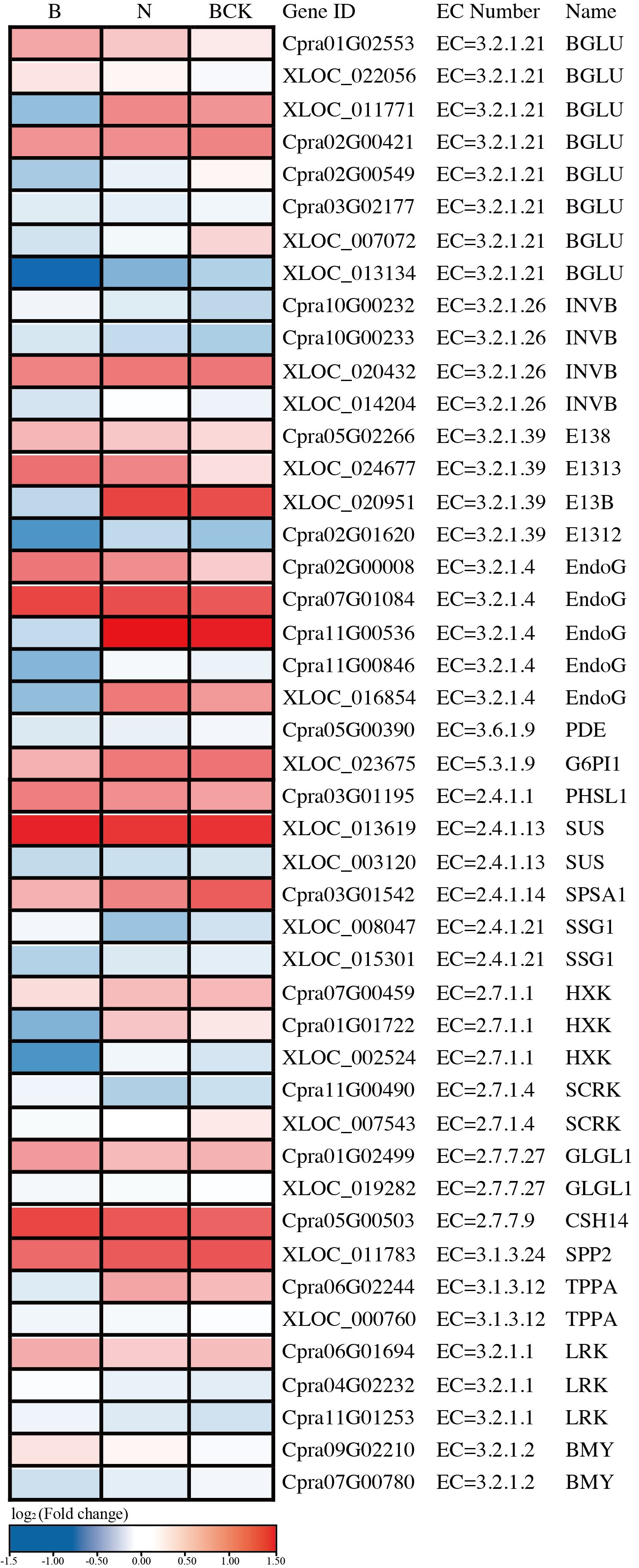

Supplement: Supplementary file 1 [file plants-13-02571-s001.zip › Figure S2. Heatmap of differential gene expression in starch and sucrose metabolism pathways (ko00500).png]
